# Characterization of a *Listeria monocytogenes* meningitis mouse model

**DOI:** 10.1186/s12974-018-1293-3

**Published:** 2018-09-07

**Authors:** Merel M. Koopmans, JooYeon Engelen-Lee, Matthijs C. Brouwer, Valery Jaspers, Wing Kit Man, Mercedes Vall Seron, Diederik van de Beek

**Affiliations:** From the Amsterdam UMC, Department of Neurology, University of Amsterdam, Amsterdam Neuroscience, Meibergdreef 9, 1105 AZ Amsterdam, The Netherlands

**Keywords:** *Listeria monocytogenes*, Mouse model, Sequence type, Histopathology, Cytokines

## Abstract

**Background:**

*Listeria monocytogenes* is a common cause of bacterial meningitis. We developed an animal model of listerial meningitis.

**Methods:**

In survival studies, C57BL/6 mice received intracisternal injections with different *L. monocytogenes* sequence type 1 (ST1) colony forming units per milliliter (CFU; *n* = 48, 10^5^, 10^6^, 10^7^, 10^8^, and 10^9^ CFU/ml). Second, mice were inoculated with 10^8^ CFU/ml ST1 and sacrificed at 6 h and 24 h (*n* = 12/group). Outcome parameters were clinical score, CFUs, cyto- and chemokine levels, and brain histopathology. Third, 84 mice were inoculated (10^9^ CFU/ml ST1) to determine optimal antibiotic treatment with different doses of amoxicillin and gentamicin. Fourth, mice were inoculated with 10^9^ CFU/ml ST1, treated with amoxicillin, and sacrificed at 16 h and 24 h (*n* = 12/group) for outcome assessment. Finally, time point experiments were repeated with ST6 (*n* = 24/group).

**Results:**

Median survival time for inoculation with 10^8^ and 10^9^ CFU/ml ST1 was 46 h and 40 h; lower doses of bacteria led to minimal clinical signs of disease. Brain levels of IL-6, IL-17A, and IFN-γ were elevated at 24 h, and IL-1β, IL-6, IL-10, IFN-γ, and TNF-α were elevated in blood at 6 h and 24 h. Histopathology showed increased meningeal infiltration, vascular inflammation of meningeal vessels, hemorrhages, and ventriculitis. In the treatment model, brain levels of IL-6 and IL-17A and blood levels of IL-6 and IFN-γ were elevated. Compared to ST6, infection with ST1 led initially to higher levels of IL-1β and TNF-α in blood and more profound neuropathological damage. At 16 h post inoculation, IL-1β, IL-10, and TNF-α in blood and IL-6, IL17A, TNF-α, and IFN-γ levels in brain were higher in ST1 compared to ST6 without differences in CFUs between STs. At 24 h, neuropathology score was higher in ST1 compared to ST6 (*p* = 0.002) infected mice.

**Conclusions:**

We developed and validated a murine model of listerial meningitis. ST1-infected mice had a more severe inflammatory response and brain damage as compared to ST6-infected mice.

**Electronic supplementary material:**

The online version of this article (10.1186/s12974-018-1293-3) contains supplementary material, which is available to authorized users.

## Background

Bacterial meningitis is a life-threatening infectious disease of the central nervous system, which is most commonly caused by *Streptococcus pneumoniae* and *Neisseria meningitidis* [1, 2]. *Listeria monocytogenes* is the third most common pathogen causing bacterial meningitis in adults, and is found in 5–10% of cases [2–4]. Listeria distributes easily in the environment and can be found in soil, ground water, and feces of animals [5, 6]. Main source for human infection is food, and it primarily affects elderly and immunocompromised persons, [7] in whom it can cause up to 40% of community-acquired bacterial meningitis cases [8, 9]. A nation-wide prospective cohort study on *L. monocytogenes* meningitis described an increasing mortality rate over time, from 17 to 36% over the past decade [10]. This trend was also reported by a French cohort study including 252 patients with listerial meningitis [7].

In the immune response neutrophils, monocytes and macrophages are activated by pro-inflammatory cytokines such as IL-1α, IL-1, IL-6, IL-12, TNF-α, and IFN-γ [11–14]. Anti-inflammatory cytokine IL-10 plays an important role in limiting immune-mediated damage and at the same time antagonizes IFN-γ activity which makes the host more vulnerable for an invasive listerial infection [14]. Several mouse and rat listeria models have been developed to study invasive *L. monocytogenes* diseases including cerebral and meningeal infection, using oral [15–17], intravenous [18], intracerebral [19–23], or intracisternal inoculation methods [24–26]. Problems with reproducibility, limited disease progression, or iatrogenic structural damage, combined with a need for a single model in which most pathological features seen in human listerial meningitis can be measured, have created the need for development of a new animal model. We developed a listerial meningitis mouse model to counter these problems and compared infection with listerial ST1 with ST6.

## Methods

Bacterial strain *L. monocytogenes* sequence type 1 (ST1) was used for the experiments, time point studies were repeated with a ST6 strain. Both strains were obtained from human positive cerebrospinal fluid (CSF) isolates stored at the Netherlands Reference Laboratory for Bacterial Meningitis (NRLBM). The isolates were grown to mid-log phase in 1–1.5 h at 37 °C in BHI to an optical density (OD600) of 0.45–0.55, then, centrifuged at 2000 rpm for 20 min at 4 °C. Supernatant was removed, and sterile 0.9% NaCl was added to yield the needed concentration. Before and after inoculation, the dose was determined by serial dilution method and plated on blood agar plates overnight at 37 °C.

Experiments were performed with eight- to ten-week-old C57BL/6 mice (Charles River Laboratories, Germany). In the non-treatment survival experiments, both sexes were used; in the non-treatment time point experiments and all treatment experiments, male C57BL/6 mice were used. The mice were kept to a controlled 12-h light/dark cycle, and food and water were provided ad libitum. All experiments were approved by the Institutional Animal Care and Use Committee of the Academic Medical Center, Amsterdam and performed according to the institution and Animal Research: Reporting of In Vivo Experiments (ARRIVE) guidelines [27].

### Mouse model of listerial meningitis: non-treatment

Survival experiments were performed to determine clinical course of disease with the aim to achieve a median lethal dose for 50% of mice (LD50) after 36–48 h. Mice were inoculated with 1 μl bacterial suspension *L. monocytogenes* ST1 into the cisterna magna using a 32-gauge needle and syringe to dispense 1–10 μl. During inoculation, mice received short-term anesthesia using 2% isoflurane (Baxter). Five inoculum sizes were tested between 10^5^ and 10^9^ CFU/ml (*n* = 6 in 10^5^ and 10^6^ CFU/ml, *n* = 12 in 10^7^ and 10^9^ CFU/ml groups). After inoculation, mice were checked according to a clinical scoring list for direct neurological deficits which could indicate puncture failure (such as occipital bleeding). If direct neurological deficits were found, mice were euthanized and excluded from the experiment. Mice were monitored every 4–6 h (starting from 12 h after inoculation), and the clinical score (Table 1) and the portion of surviving mice in each group was determined up to 90% mortality [28]. Mice were sacrificed by intraperitoneal injection (i.p.) with dexmedetomidine (0.3 mg/kg) in combination with ketamine (190 mg/kg) when a clinical score of ≥ 15 was reached. Subsequently, time point experiments were performed (*n* = 12 per time point) with 10^8^ CFU/ml *L. monocytogenes* ST1 or ST6 which were compared to a control group (*n* = 6) receiving 1 μl 0.9% NaCl intracisternally. At 6 and 24 h post-inoculation, mice were sacrificed, and blood, CSF, and organs were collected, processed, and stored [28, 29].

**Table 1:** Clinical scoring list for bacterial meningitis mouse model

Parameter	Value	Weighted score	Max. score
Weight loss	Normal 5%	0	
	5–10%	1	
	10–15%	2	
	15–20%	3	
	20–25%	4	4
Activity	Normal	0	
	Increased/aggressive	1	
	Mildly diminished	1	
	Diminished	2	
	Severely diminished	3	3
Condition	Normal, does not lay on back	0	
	Upright within 5 s	2	
	Upright within 30 s	4	
	Does not turn upright	6	6
Coat	Normal	0	
	Diminished grooming	1	
	Soiled	1	
	Piloerection	1	3
Posture	Normal	0	
	Slightly hunched back	1	2
	Severe hunched back	2	
Eyes	Normal	0	
	Protruding	1	
	Closed eyelids	1	
	Discharge	1	4
Respiration rate (per min)	> 150	0	
	100–150	1	
	75–100	2	
	50–75	3	
	< 50	4	4
Breathing	Irregular	2	
	Labored	2	4
Neurological examination	Normal	0	
	Coordination problem	2	
	Paresis/paralysis	2	
	Epileptic seizure	2	
	Status epilepticus	6	10
Humane endpoints	Total score ≥ 15		
	Status epilepticus		
	≥ 2 seizures in 15 min		
	Hemiparalysis		
	≥ 25% weight loss		

### Mouse model of listerial meningitis: treatment

Three survival experiments with 84 male mice (12 mice per subgroup) were performed to test dosage and frequency of intraperitoneal amoxicillin treatment, and the potentially beneficial effect of adding intraperitoneal gentamicin, as is the preferred antibiotic treatment used in human listerial meningitis [30]. After each experiment, brains of one or two surviving mice per subgroup were harvested to measure bacterial outgrowth. In the first experiment, mice were inoculated with 10^9^ CFU/ml *L. monocytogenes* ST1 and treated 16 h post-inoculation with 50 or 100 mg/kg amoxicillin every 24 h. In the second experiment, higher doses (100 vs. 200 mg/kg/24 h amoxicillin) and shorter treatment interval (100 mg/kg amoxicillin every 12 h vs. every 24 h) was tested, starting 16 h post-inoculation. To determine the effect of additional gentamicin, 20 mg/kg/24 h of gentamicin was administered concomitant to 100 mg/kg/24 h amoxicillin 16 h post-inoculation compared to 100 mg/kg/24 h amoxicillin only in mice who were inoculated with 10^8^ CFU/ml. In the time point treatment experiments, mice were inoculated with 10^9^ CFU/ml *L. monocytogenes* ST1 or ST6 per strain. Twelve mice were sacrificed 16 h after infection, and 12 mice were treated with 100 mg/kg amoxicillin i.p. 16 h after infection and sacrificed after 24 h.

### Scoring, harvesting, and cytokine analyses

Clinical scoring was performed by two observers according to a previously developed scoring list for a pneumococcal meningitis mouse model (Table 1) [28]. Each scoring parameter ranges from zero, corresponding to no abnormalities, to a variable maximum score. Animals reaching humane endpoint (HEP) criteria were humanely killed. After anesthetizing the mice, cardiac puncture was performed for blood collection, and intracisternal puncture for CSF collection. Brain, spleen, liver, and lungs were harvested and processed as described previously [28]. Supernatant, plasma, and CSF were stored at − 80 °C until further use. Cytokine analyses were performed using Luminex technology Bio-plex Pro Mouse Cytokine 6-plex Assay (Bio-Rad Laboratories, Veenendaal, the Netherlands). We analyzed levels of IL-1β, IL-6, IL-10, IL-17A, TNF-α, and IFN-γ to include early and late pro- and anti-inflammatory cytokines. For cytokine values below the lower limit of detection (LLOD), the LLOD was used in the calculation of median and interquartile range. Histopathology was performed on the left hemisphere of the brain. Brain was fixed in 4% paraformaldehyde and paraffin embedded in seven coronal plaques. In all mice, a hematoxylin and eosin (H and E) staining and Gram staining were performed. Histopathology was scored (blinded) in six categories by a neuropathologist as previously described. An additional table shows this in more detail (Additional file 1) [29].

Comparison of survival curves between groups within each model was calculated using the log-rank test. Clinical scores were compared using a linear mixed model, assuming an exponential and group-specific time effect. Comparisons of cytokine levels between groups were calculated using the Mann–Whitney U test, and for dichotomous variables in histopathology scoring, the Fisher’s exact test was used. All statistical tests were two-tailed, and a *p* value of < 0.05 was considered to be significant.

## Results

### Non-treatment model

**Fig. 1:**
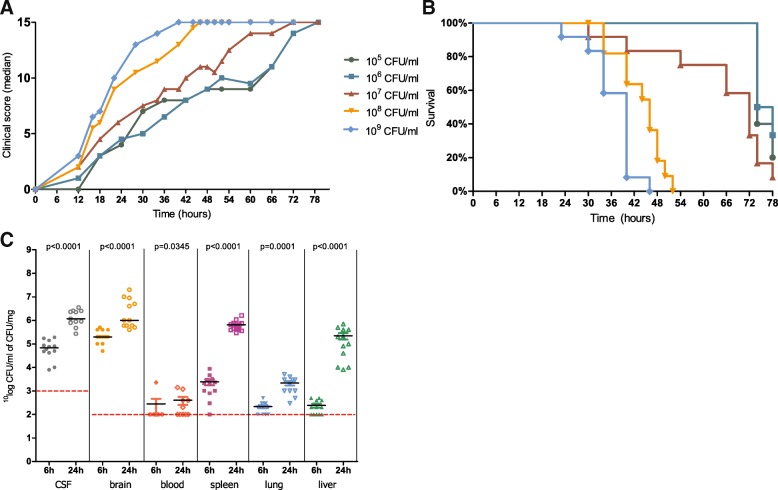
Clinical score (**a**) and Kaplan–Meier curves (**b**) after intracisternal injection with ST1. Bacterial outgrowth after inoculation with 10^8^ CFU/ml ST1 (**c**) “**---**” lower limit of detection (LLOD). h = hours

In the survival study, mice inoculated with 10^5^, 10^6^ and 10^7^ CFU/ml *L. monocytogenes* ST1 showed minimal clinical signs of disease limited to diminished grooming and/or a slightly hunched back. They had to be sacrificed based on weight loss, ≥ 25% according to the predefined endpoint in the clinical scoring (Fig. 1a), and therefore considered not representable for a listerial meningitis model. Mice inoculated with 10^8^ CFU/ml *L. monocytogenes* ST1 showed signs of illness 12 h after inoculation, consisting of discharge in eyes, a slightly hunched back, mildly diminished activity and/or piloerection (median clinical score of 2; interquartile range [IQR 2–2]), and had a median survival time of 46 h (IQR 34–52 h; Fig. 1b). Mice inoculated with 10^9^ CFU/ml *L. monocytogenes* ST1 had signs of illness 12 h after inoculation (median clinical score of 3 [IQR 3–4]), and a median survival time of 40 h (IQR 23–46 h). The concentration of 10^8^ CFU/ml was determined to be the optimal inoculum size for the non-treatment model. There was no difference in male or female mice in survival. An additional figure shows this in more detail (Additional file 2).

After intracisternal injection of 10^8^ CFU/ml *L. monocytogenes* ST1, CFUs increased significantly over time in CSF, blood, and collected organs (Fig. 1c). Median *L. monocytogenes* concentration in the CSF was 6.8 × 10^4^ CFU after 6 h and 1.2 × 10^6^ CFU/ml after 24 h. In the brain homogenates, median titres were 2.0 × 10^5^ after 6 h and 1.0 × 10^6^ CFU/mg after 24 h. In the spleen, lungs, and liver homogenates, median bacterial titres were 2.1 × 10^3^, 2.0 × 10^2^, and 2.0 × 10^2^ CFU/mg after 6 h and 4.9 × 10^5^, 2.0 × 10^3^, and 1.5 × 10^5^ CFU/mg after 24 h respectively. Median titre in blood at 6 h was under the LLOD (< 1.0 × 10^2^ CFU/ml) and 1.5 × 10^2^ CFU/ml after 24 h.

**Fig. 2:**
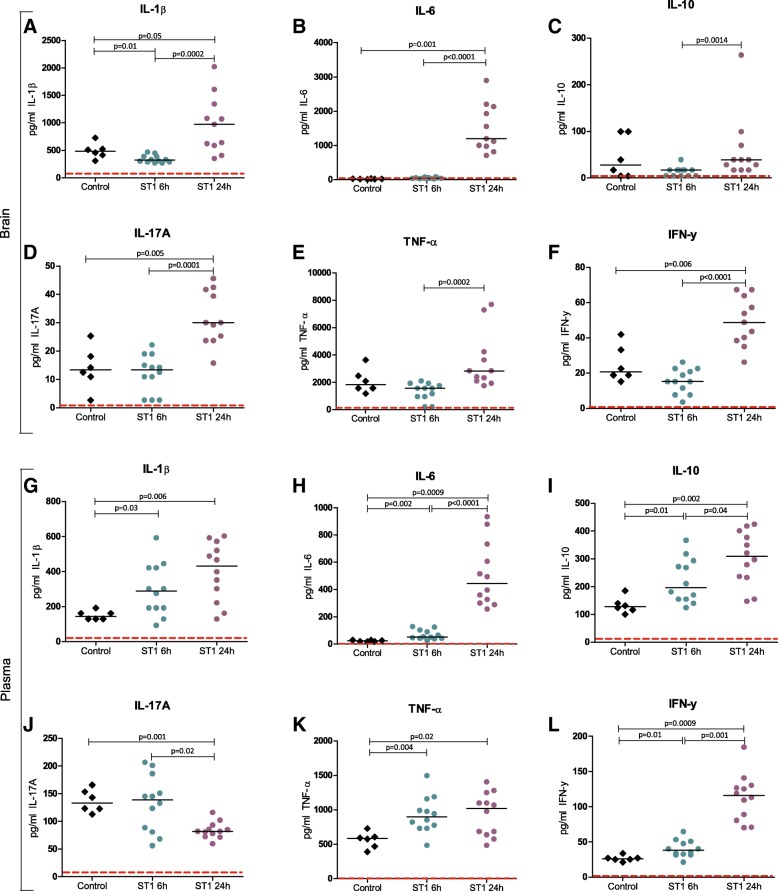
Brain (**a**–**f**) and plasma (**g**–**l**) levels of cytokines in negative controls and mice infected with 10^8^ CFU/ml ST1. “**---**” lower limit of detection (LLOD) in picogram per milliliter: **a** IL-1β = 66.887, **b** IL-6 = 1.191, **c** IL-10 = 4.220, **d** IL-17A = 2.732, **e** TNF-α = 207.460, **f**. IFN-γ = 0.876, **g** IL-1β = 12.664, **h** IL-6 = 1.173, **i** IL-10 = 3.620, **j** IL-17A = 2.720, **k** TNF-α = 3.943, **l** IFN-γ = 0.786. h = hours

Luminex analyses showed similar cytokine levels in the brain at the 6-h time point compared to negative controls, except for IL-1β in which levels were lower compared to the controls. Levels of IL-6, IL-17A, and IFN-γ were elevated in brain homogenates at the 24-h time point in infected mice compared to controls (Fig. 2), and all increased after 24 h compared to the 6-h time point. Plasma levels of all measured cytokines with the exception of IL-17A were elevated at both time points compared to the controls, and IL-6, IL-10, and IFN-γ increased between 6 h and 24 h (Fig. 2).

**Fig. 3:**
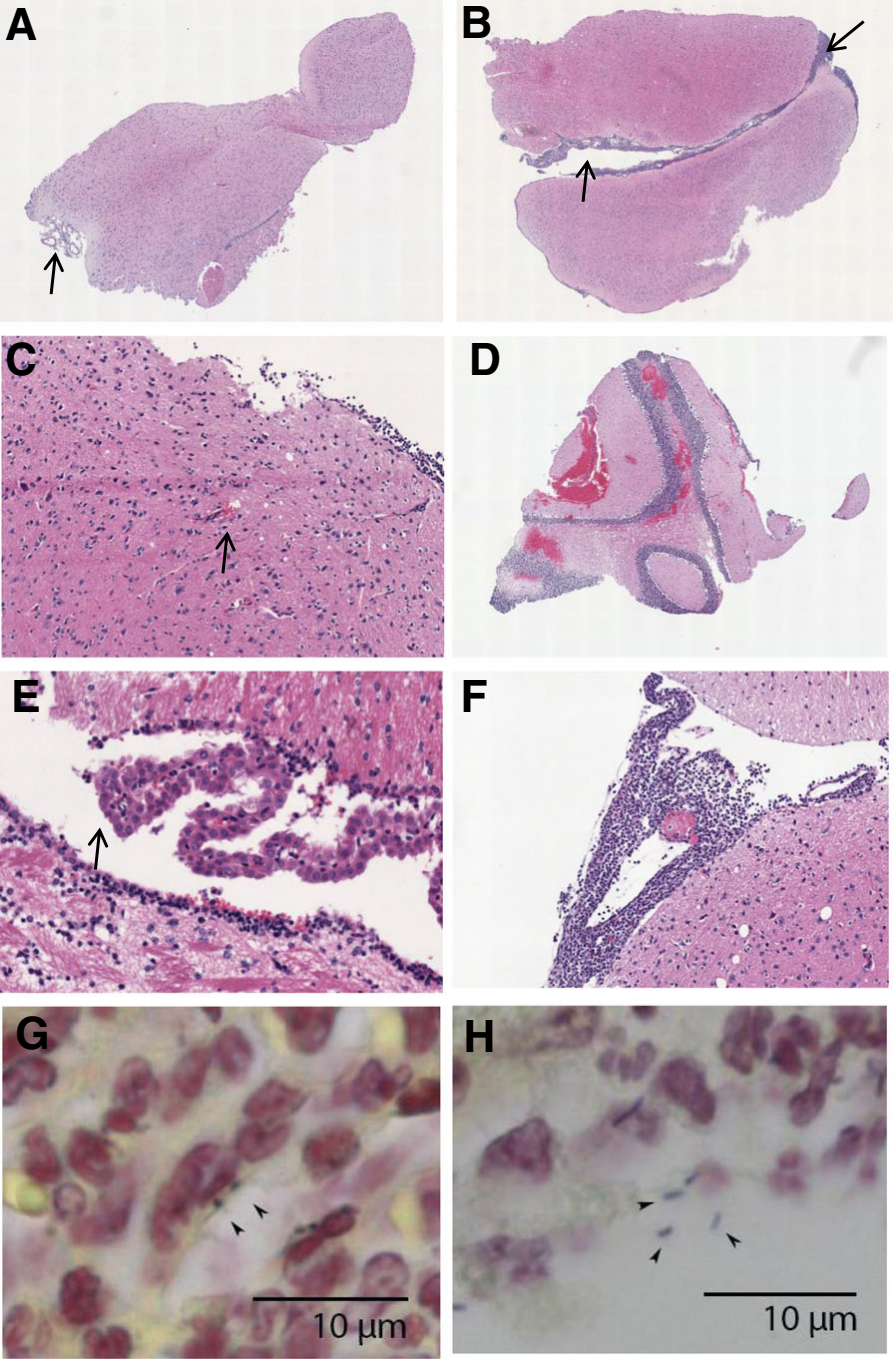
Histopathology in listerial meningitis mice model. Mild meningeal infiltration (**a**, score 1), severe meningeal infiltration (**b**, score 3), focal and meningeal bleeding (**c**, score 1), bleeding (**d**, score 3) ventriculitis (**e**, score 3), focal thrombosis (**f**, score 1). Gram staining of meninges demonstrating intra-(**g**) and extracellular (**h**) bacteria

Histopathology showed increased brain damage between *t* = 6 and *t* = 24 in the non-treatment model. Median pathology score increased from 2 [IQR 1–4] to 7 [IQR 6–9] (*p* = 0.001, an additional table shows this in more detail Additional file 3) due to increase from mild focal meningeal infiltration (Fig. 3a) to severe meningeal infiltration (Fig. 3b), and meningeal vascular inflammation from one mouse (8%) 6 h post-inoculation to 12 mice (100%) 24 h post-inoculation. Focal small parenchymal and subarachnoid bleedings were present in 6 of 12 mice at 6 h (50%), and developed into multiple parenchymal and subarachnoid hemorrhages at 24 h (in 58%, Fig. 3c). Ventriculitis was present in 17% of mice at 6 h to 42% at 24 h.

### Treatment model

Treatment time point was chosen at 16 h post-infection based on clinical course of disease in the survival experiments in mice inoculated with 10^9^ CFU/ml bacteria. Treatment with 100 mg/kg/24 h of amoxicillin was superior to 50 mg/kg/24 h of amoxicillin in survival (median survival 70 h vs. 46 h, *p* < 0.001; an additional figure shows this in more detail Additional file 4a). Further experiments showed survival did not improve by treating mice with 200 mg/kg/24 h or 100 mg/kg/12 h of amoxicillin or adjuvant 20 mg/kg/24 h of gentamicin compared to 100 mg/kg/24 h of amoxicillin, and no significant differences were found in clinical scoring (Additional files 2 and 4). In all survival experiments, brain homogenates from mice that survived till the end of the experiment (70 h post-inoculation) showed bacterial outgrowth. Decreasing the inoculum size to 10^8^ CFU/ml did not improve clearance of bacteria. An additional table shows this in more detail (Additional file 5).

In the time point study, bacterial titres in all collected fluids and organs decreased between 16 h and 24 h after treatment with 100 mg/kg/24 h of amoxicillin. Sixteen hours post-inoculation median bacterial titre in CSF was 4.3 × 10^6^ CFU/ml, and decreased after treatment with amoxicillin to 5.2 × 10^5^ CFU/ml at 24 h. An additional figure shows this in more detail (Additional file 4). Median bacterial concentration in the brain homogenates was 6.4 × 10^7^ after 16 h and 1.0 × 10^6^ CFU/mg after 24 h, and in blood, spleen, lungs, and liver homogenates, median bacterial titres were 2.0 × 10^3^, 3.3 × 10^6^, 8.2 × 10^3^, and 4.1 × 10^4^ CFU/mg after 16 h and 1.6 × 10^2^, 1.3 × 10^5^, 2.3 × 10^3^, and 2.2 × 10^4^ CFU/mg after 24 h respectively.

**Fig. 4:**
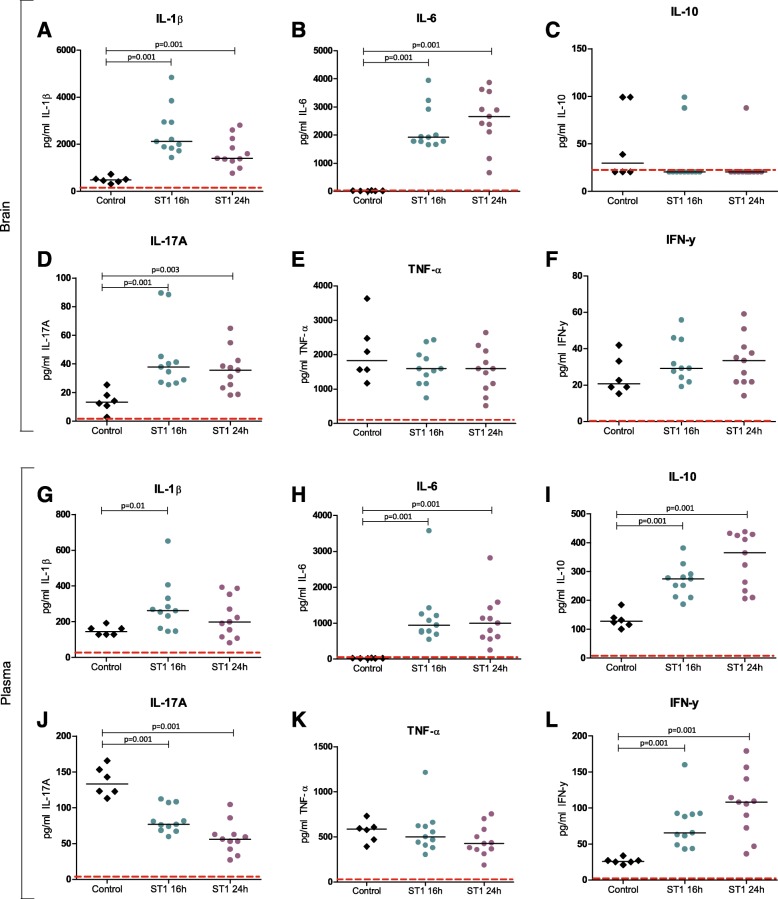
Brain (**a**–**f**) and plasma (**g**–**l**) levels of cytokine in negative controls and mice infected with 10^9^ CFU/ml ST1 treated with 100 mg/kg amoxicillin at 16 h. “**---**” lower limit of detection (LLOD) in picogram per milliliter: **a** IL-1β = 3.070, **b** IL-6 = 1.297, **c** IL-10 = 20.477, **d** IL-17A = 2.858, **e** TNF-α = 60.390, **f** IFN-γ = 0.724, **g** IL-1β = 11.592, **h** IL-6 = 1.157, **i** IL-10 = 4.482, **j** IL-17A = 2.702, **k** TNF-α = 3.618, **l** IFN-γ = 0.675. h = hours

Cytokine measurements showed elevated IL-1β, IL-6, and IL-17A concentration in ST1-infected mice compared to negative controls in both time points (Fig. 4). Luminex of plasma in the treatment model showed elevated levels of IL-6, IL-10, and IFN-γ and decreased levels of IL-17A at both time points. IL-1β was elevated at the 16-h time point in ST1-infected mice compared to controls (Fig. 4).

Overall, histopathological score was similar between 16- and 24-h time points (median score 7 [IQR 5–8] vs. 9 [IQR 8–12]). An additional table shows this in more detail (Additional file 3). Meningeal infiltration and meningeal vascular inflammation were present in all mice. As seen in the non-treatment model, bleeding and ventriculitis were frequently present. Hemorrhages were found parenchymal or subarachnoidal, and were categorized as small and focal, 16 h post-inoculation in 73% of mice and as large hemorrhages at multiple locations in 91% after 24 h.

### Bacterial strain ST1 vs. ST6

In the non-treatment time point experiments, clinical score and bacterial outgrowth of ST6 in CSF, blood, and collected organs were comparable to ST1. Cytokine and chemokine levels in brain homogenates measured at similar time points did not differ between ST1 and ST6. In plasma, IL-1β and TNF-α of both STs were significantly elevated 6 h post-inoculation, and ST1 24 h post-inoculation compared to the negative controls. At *t* = 24, there was a significant difference between strains in median IL-1β levels, ST1 432 pg/ml [IQR 282–533 pg/ml] and ST6 192 pg/ml [IQR 137–307 pg/ml] (*p* = 0.04), and at both time points, there was a significant difference in TNF-α levels between ST1 and ST6 (TNF-α *t* = 6; ST1 900 pg/ml [IQR 766–1034] vs. ST6 746 pg/ml [IQR 639–812] (*p* = 0.03) and at *t* = 24; ST1 1022 pg/ml [IQR 674–1137] vs. 470 pg/ml [IQR 370–746], *p* = 0.045). Histopathology scores in ST1- and ST6-infected mice were similar in terms of meningeal infiltration, severity of ventriculitis, thrombosis, and increase of meningeal vascular inflammation over time. Six hours post-inoculation, focal small parenchymal and meningeal bleeding was present in 6 of 12 ST1 mice (50%), while it was only seen in one of the 12 ST6-infected mice (8%, *p* = 0.03), but overall pathology scores were similar between ST1- and ST6-infected mice at 24 h.

There were no significant differences in clinical score and bacterial outgrowth in collected fluids or organs between ST1 and ST6 in both non-treatment and treatment model. An additional figure shows this in more detail (Additional file 6). In the treatment model, five ST6 mice had to be euthanized (three because of puncture failure and two because of wounds after fighting). However, brain levels of IL-6, IL17A, TNF-α, and IFN-γ in ST1-inoculated mice compared to ST6 16 h. An additional table shows this in more detail (Additional file 7). In plasma, IL-1β levels were higher in ST1-inoculated mice at both time points compared to ST6-infected mice, and IL-10 and TNF-α levels were higher at the 24-h time point in ST1-infected mice. Overall pathology score was higher at 24 h in ST1-infected mice (median score 9; [8–11, 31]) compared to ST6-inoculated mice (median score 7; [6, 7], *p* = 0.002). This was mainly driven by increased meningeal infiltration, ventriculitis, and hemorrhages in ST1-infected mice.

## Discussion

We developed and validated a murine model of listerial meningitis*.* We used *Listeria* sequence types [18, 32] that commonly cause invasive disease [18, 31]. Previously described intracisternally inoculation mice and rat studies used a serotype 4b strain with unknown sequence type [24–26]; intracerebral inoculation mice studies [22, 23] used a less virulent laboratory EGD strain compared to the ST1 and ST6 strains [18, 33]. We used intracisternal inoculation aiming for a reproducible meningitis model. Previous studies using oral and intravenous inoculation reported difficulties with respect to neuro-invasion reproducibility [15–17, 34–36], while intracerebral injection primarily causes cerebritis. Intracerebral inoculation methods have been used successfully previously to study the role of macrophage inflammatory protein and TNF-α in listerial meningitis [22, 23]. Intracisternal inoculation has been used to study heat production [24], compare effectiveness of antibiotics [26], and the role of reactive oxygen and nitric oxide in listeria growth [25]. Our model allows evaluation of multiple features including bacterial growth, host immune response, clinical severity, and histopathological damage.

Main histopathological characteristics of listerial meningitis were meningeal inflammation, ventriculitis, and abscesses. This is in line with previous studies [37, 38]. We also observed a high rate of cerebral hemorrhages, an uncommon feature in human listerial meningitis (2% of cases) [10]. This is consistent with the observed difference in pneumococcal meningitis in a human and mouse model [28, 39]. In human bacterial meningitis, it has been suggested that dysregulation of coagulation and fibrinolytic pathways, vascular endothelial cell swelling, and vasculitis plays a role in the pathophysiology of hemorrhages [40–44].

IL-1β, IL-6, IL-10, IL-17A, TNF-α, and IFN-γ mediated the hosts immune response against *L. monocytogenes*. Previous listerial mouse models showed that monocyte recruitment to the brain is triggered by pro-inflammatory cytokines in particular IFN-γ- , TNF- , and IL-6-related immune response [45, 46]. These cytokines and IL-1β and IL-17A are able to mobilize phagocytes and activate other cytokines [11–14, 46–54], whereas IL-10 limits the immune-mediated injury; nonetheless can increase severity of *L. monocytogenes* disease by reducing the immune response [55–57]. Since IL-6 and IFN-γ were elevated in the brain and blood of both our treatment and non-treatment mouse models, these cytokines are relevant outcome measures in our model to study changes in the inflammatory response. An interesting aspect of IFN-γ is its ambiguous role in listerial infections. It is known for its protective and controlling role in the early immune response, though it seems to promote susceptibility for *L. monocytogenes* later on. Study models with interferon-deficient mice showed protective effects during systemic listerial infections [58–60]. In CSF of patients with listerial meningitis, elevation of IFN-γ, IFN-α2, and interferon-related cytokines IL-18, CX3CL1, and CCL20 were associated with an unfavorable outcome [61]. The use of amoxicillin, a bacteriolytic antibiotic, in our model did not lead to a significant increase in cytokine levels after therapy, as observed in other experimental models of bacterial meningitis also using bacteriolytic antibiotic.

Infection with *Listeria* strain of the ST1 type led to a more rigorous inflammatory response and more brain damage as compared to infection with ST6. Both STs have been marked as hypervirulent strains with a tropism for neuro-invasion [18]. ST1 has been among the most common genotypes causing listerial meningitis in the Netherlands over the last 25 years [31]. ST6 has been emerging over the last years and has been associated with an increasing rate of unfavorable outcome among adults with listerial meningitis, from 27 to 61% over a 14-year period [10]. The increased incidence of ST6 listerial meningitis in the Netherlands has been associated with the introduction of a novel plasmid, carrying the efflux transporter emrC [62]. Although speculative, differences in virulence between ST1 and ST6 found in our model could be explained by (i) degree of cell-to-cell spread from infected phagocytes to endothelial cells [63, 64]; (ii) the interaction with macrophages, neutrophils, and subsequently the cytokine signaling [65]; (iii) the proportion of *Listeria* bacteria residing in the brain parenchyma rather than extracellularly in the CSF, and thereby causing different degrees of histopathological damage [66]; (iv) degree of expression of the specific neuro-invasive internalin InlF and its binding to the filament protein vimentin [67, 68]; (v) presence of certain genetic elements in the bacteria such as LIPI-3 (in both ST1 and ST6) [69], LGI 2 (found in ST1) [70], or pLMST6 (found in ST6) [62]; (vi) other yet unknown factors influencing and differentiating the virulence of *L. monocytogenes* strains. Since ST1 and ST6 are clinically relevant strains, these unknown factors should be investigated and might help to unravel the pathophysiology of *L. monocytogenes*.

Our model has several limitations, of which some are inherent to the use of modeling of human disease in animals. First, we infected mice by inoculating directly into the cisterna magna, while the route of infection in humans mainly is through the digestive system. However, meningitis is difficult to evoke unless bacteria are injected directly intracranial, partially because animals tend to die due to systemic illness before meningitis develops [71]. Furthermore, the amount of bacteria reaching the brain cannot be controlled using digestive tract or intravenous inoculation. Second, in the treatment survival experiments, we observed that *Listeria* could be cultured from the murine brains despite high doses antibiotic treatment. This can be explained as the pathogen is intracellular and has a relatively slow growth rate. Patients with listerial meningitis are therefore treated for at least 3 weeks. To make sure we did not use insufficient dosage or type of antibiotics, we increased the dose and frequency of the amoxicillin and added gentamycin, but these changes did not influence outcome or bacterial outgrowth at the end of the experiment. Therefore, we feel that we achieved an optimal amoxicillin dose to perform the experiments with. We did observe that bacterial counts decreased in all treated mice. It could be argued that other antibiotics with a previously suggested effect and/or had a synergism in treatment of listerial meningitis should have been tested [30]. However, amoxicillin with or without gentamicin is the most commonly used treatment in human listerial meningitis, and therefore testing other antibiotics is beyond the scope of this article.

## Conclusions

The listerial meningitis mouse model provides an experimental setting of listerial meningitis with multiple outcome parameters. Similar model set up in pneumococcal meningitis has proven to be useful in exploring inflammatory hypotheses in pneumococcal meningitis [72–74]. Integration of these pathological features in a single model is a valuable tool in the further investigation of both pathophysiological and therapeutic intervention studies in listerial meningitis.

## Additional files

Additional file 1: This table shows a histopathological scoring method of brain tissue in bacterial meningitis mouse model which has been used in this study and previously has been used in a pneumococcal meningitis model. (DOC 48 kb)

Additional file 2: Kaplan-Meier survival curve (A) in male and female mice (24 mice/group). Clinical score of the treatment survival experiments (12 mice/ group) inoculated with 10^9^ CFU bacteria and treated with antibiotics. Abbreviation; h = hours (PDF 30 kb)

Additional file 3: This table shows histopathological scoring of brain tissue in listerial meningitis time point studies with *L. monocytogenes* ST1 and ST6 strains. Results are presented based on number of mice and on median pathology score. (DOC 82 kb)

Additional file 4: Kaplan-Meier survival curves in treatment survival experiments inoculated with 10^9^ CFU/ml (A and B) and with 10^8^ CFU/ml (C) and bacterial outgrowth after inoculation with 10^9^ CFU/ml *L. monocytogenes* ST1 and amoxicillin treatment (D). **---** lower limit of detection, Abbreviation; h = hours. (PDF 44 kb)

Additional file 5: This table shows bacterial outgrowth in brain homogenate in mice infected with *L. monocytogenes* ST1 and treated with antibiotics during survival experiments (70 h post inoculation). Every bacterial count represents one mouse. (DOC 51 kb)

Additional file 6: (A) Median clinical score in ST1 and ST6 inoculated mice in the non-treatment model with interquartile ranges, (B) Bacterial outgrowth in the non-treatment model ST1 vs. ST6 6 h after inoculation. Titres are expressed per mice and with median CFU/ml or CFU/mg. (PDF 38 kb)

Additional file 7: This table shows the brain and plasma levels of cytokines in mice infected with 10^9^ CFU/ml *L. monocytogenes* ST1 or ST6 at time points 16 and 24 h and treated with 100 mg/kg/24 h amoxicillin after 16 h. (DOC 64 kb)

## Abbreviations

ARRIVE: Animal Research: Reporting of In Vivo Experiments
CC: Clonal complex
CFU: Colony forming units
CSF: Cerebrospinal fluid
H and E: Hematoxylin and eosin staining
HEP: Humane endpoint
i.p.: Intraperitoneal
IQR: Interquartile range
LD50: Lethal dose for 50% of mice
LLOD: Lower limit of detection
NRLBM: Netherlands Reference Laboratory for Bacterial Meningitis
OD: Optical density
ST1: Sequence type 1
