# Supplementary material for: Characterization of a Listeria monocytogenes meningitis mouse model
Source: J Neuroinflammation. 2018 Sep 7;15:257. doi: 10.1186/s12974-018-1293-3 (PMC6128981; doi:10.1186/s12974-018-1293-3)
Supplement: Supplementary file 1 — This table shows a histopathological scoring method of brain tissue in bacterial meningitis mouse model which has been used in this study and previously has been used in a pneumococcal meningitis model. (DOC 48 kb) [file 12974_2018_1293_MOESM1_ESM.doc]

**Additional file 1.** Histopathological scoring method of brain tissue in bacterial meningitis mouse model.

| **Main category** | **Subcategory** | Score | | | |
| --- | --- | --- | --- | --- | --- |
|  |  | **0** | **1** | **2** | **3** |
| Meningeal infiltration |  | Absent | Focal mild infiltration | Multifocal mild **or** focal severe infiltration | Multifocal severe infiltration |
| Vascular inflammation | Large meningeal artery inflammation  Small parenchymal vessel inflammation | Absent | Focal mild subendothelial infiltration /reactive changes | Multifocal mild subendothelial infiltration /reactive changes **or**  focal severe vascular wall infiltration with obstruction and/or destruction of vessels | Multifocal severe vascular wall infiltration with obstruction and/or destruction of vessels |
| Ventriculitis |  | Absent | A few inflammatory cells in the ventricle | Groups of inflammatory cells in the ventricle with/without ependymal infiltration | Extension of inflammatory cells into the periventricular tissue |
| Parenchymal damage | Infarction, Hemorrhage | Absent | Focal small damage | Multifocal small **or**  focal large damage | Multifocal large damages |
| Thrombosis |  | Absent | Focal mild with partial obstruction of vascular lumen | Multifocal mild with partial obstruction of vascular lumen **or**  focal severe with complete obstruction of vascular lumen and destruction of vessel wall | Multifocal severe with complete obstruction of vascular lumen and destruction of vessel wall |
| Abscess |  | Absent | Focal small damage | Multifocal small **or**  focal large damage | Multifocal large damages |
