# Supplementary figures and images for: Characterization of a Listeria monocytogenes meningitis mouse model
Source: J Neuroinflammation. 2018 Sep 7;15:257. doi: 10.1186/s12974-018-1293-3 (PMC6128981; doi:10.1186/s12974-018-1293-3)

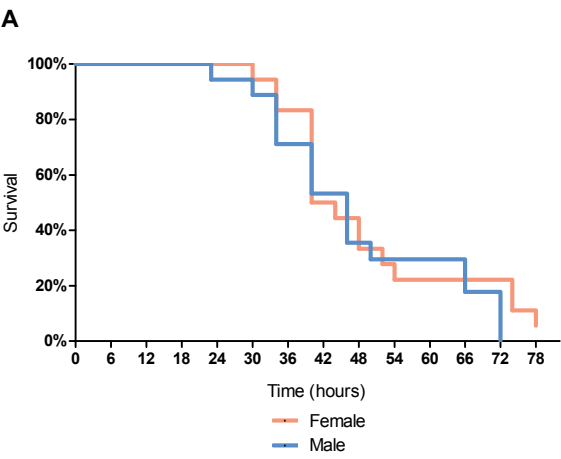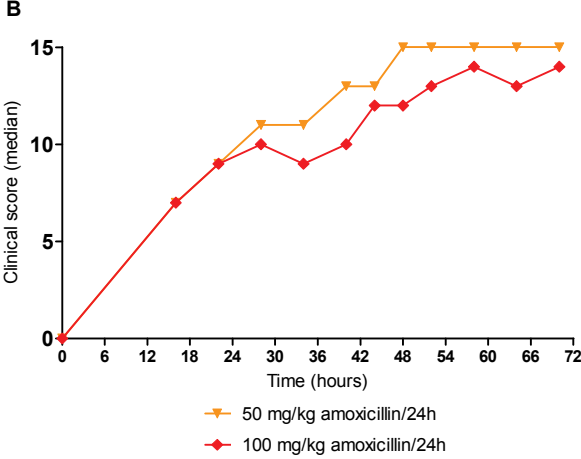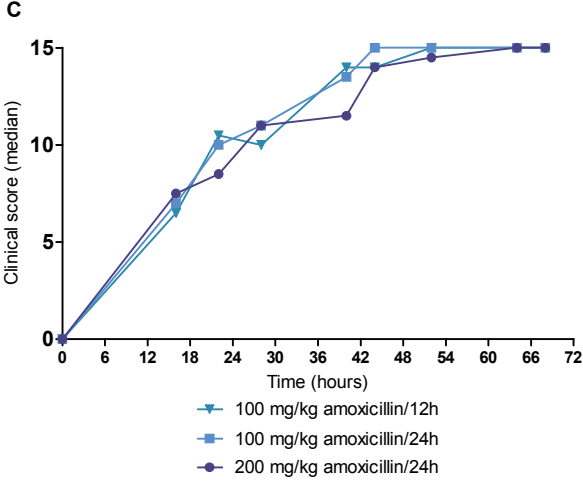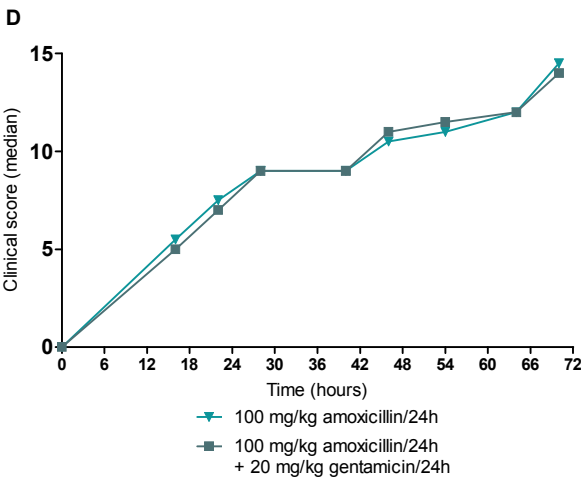

Supplement: Supplementary file 2 — Kaplan-Meier survival curve (A) in male and female mice (24 mice/group). Clinical score of the treatment survival experiments (12 mice/ group) inoculated with 109 CFU bacteria and treated with antibiotics. Abbreviation; h = hours (PDF 30 kb) [file 12974_2018_1293_MOESM2_ESM.pdf]

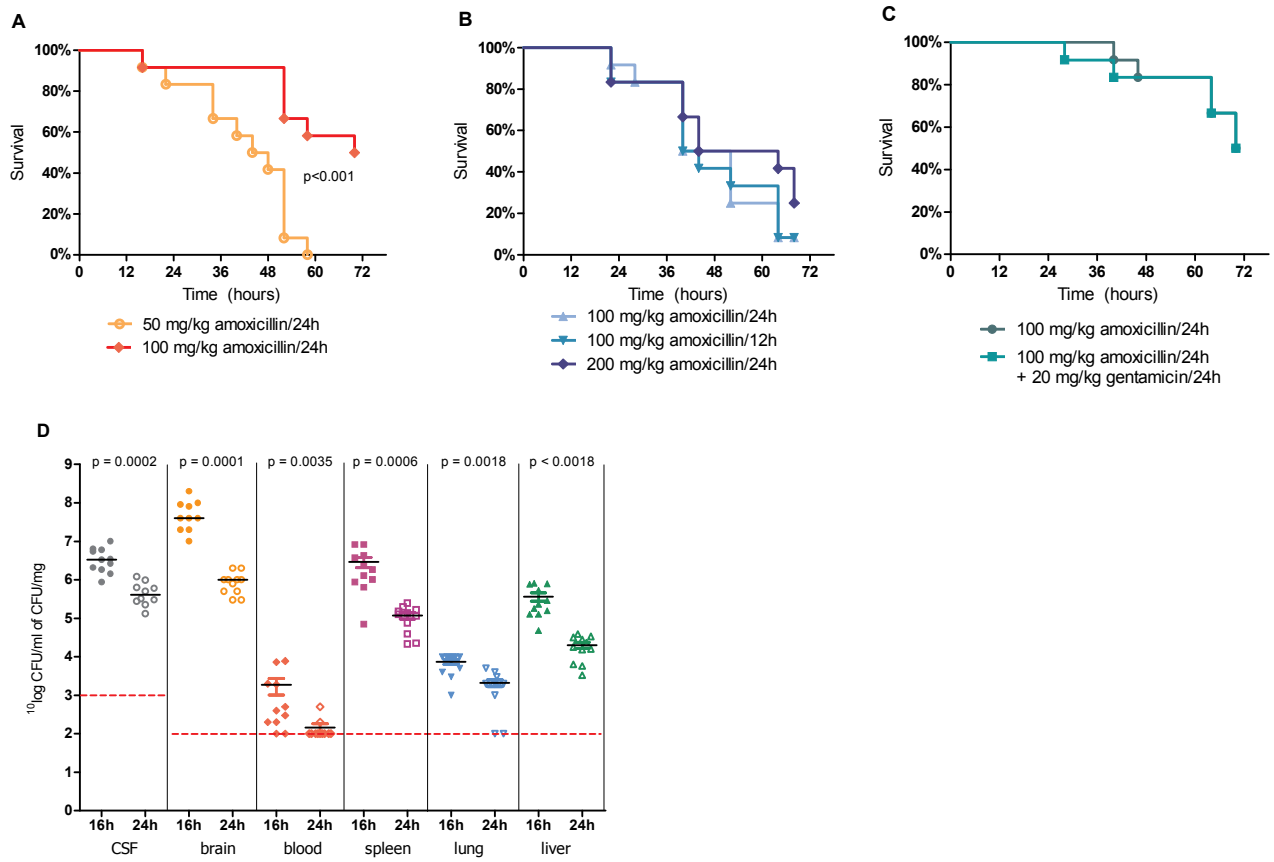

Supplement: Supplementary file 4 — Kaplan-Meier survival curves in treatment survival experiments inoculated with 109 CFU/ml (A and B) and with 108 CFU/ml (C) and bacterial outgrowth after inoculation with 109 CFU/ml L. monocytogenes ST1 and amoxicillin treatment (D). --- lower limit of detection, Abbreviation; h = hours. (PDF 44 kb) [file 12974_2018_1293_MOESM4_ESM.pdf]

A

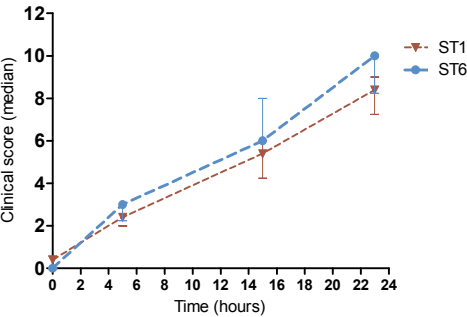

B

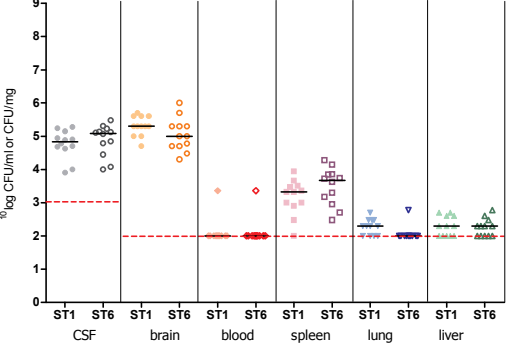

Supplement: Supplementary file 6 — (A) Median clinical score in ST1 and ST6 inoculated mice in the non-treatment model with interquartile ranges, (B) Bacterial outgrowth in the non-treatment model ST1 vs. ST6 6 h after inoculation. Titres are expressed per mice and with median CFU/ml or CFU/mg. (PDF 38 kb) [file 12974_2018_1293_MOESM6_ESM.pdf]
