# Supplementary material for: Characterization of a Listeria monocytogenes meningitis mouse model
Source: J Neuroinflammation. 2018 Sep 7;15:257. doi: 10.1186/s12974-018-1293-3 (PMC6128981; doi:10.1186/s12974-018-1293-3)
Supplement: Supplementary file 3 — This table shows histopathological scoring of brain tissue in listerial meningitis time point studies with L. monocytogenes ST1 and ST6 strains. Results are presented based on number of mice and on median pathology score. (DOC 82 kb) [file 12974_2018_1293_MOESM3_ESM.doc]

| **A** | **Meningeal infiltration** | | | **Vascular inflammation** | | | **Ventriculitis** | | | **Bleeding** | | | **Thrombosis** | | | | **Abscess** | | |  | | |  |
| --- | --- | --- | --- | --- | --- | --- | --- | --- | --- | --- | --- | --- | --- | --- | --- | --- | --- | --- | --- | --- | --- | --- | --- |
|  | **ST1**  **n (%)** | **ST6**  **n (%)** | **p-value** | **ST1**  **n (%)** | **ST6**  **n (%)** | **p-value** | **ST1**  **n (%)** | **ST6**  **n (%)** | **p-value** | **ST1**  **n (%)** | **ST6**  **n (%)** | **p-value** | **ST1**  **n (%)** | **ST6**  **n (%)** | **p-value** | | **ST1**  **n (%)** | **ST6**  **n (%)** | **p-value** |  | | | |
| **Non-treatment model** | | | | | | | | | | | | | | | | | | | |  | | | |
| **t^a^=6** | 12 (100) | 12 (100) | - | 1 (8) | 1(8) | - | 2 (17) | 0 (0) | - | 6 (50) | 1 (8) | 0,03 | 0 (0) | 0 (0) | - | | 3 (25) | 2 (17) | - |  | | | |
| **t=24** | 12 (100) | 12 (100) | - | 12 (100) | 11 (92) | - | 5 (42) | 7 (58) | - | 7 (54) | 6 (50) | - | 4 (33) | 5 (42) | - | | 4 (33) | 3 (25) | - |  | | | |
| **p-value** | - | - |  | <0,001 | <0,001 |  | - | 0,002 |  | - | 0,03 |  | 0,03 | 0,01 |  | | - | - |  |  | | | |
| **Treatment model** | | | | | | | | | | | | | | | | | | | |  | | | |
| **t=16** | 11 (100) | 8 (100) | - | 11 (100) | 8 (100) | - | 8 (73) | 2 (25) | 0,04 | 8 (73) | 8 (100) | - | 1 (9) | 1 (13) | - | | 1 (9) | 3 (38) | - |  | | | |
| **t=24** | 11 (100) | 7 (100) | - | 11 (100) | 7 (100) | - | 9 (82) | 5 (71) | - | 10 (91) | 7 (100) | - | 3 (27) | 2 (29) | - | | 1 (9) | 2 (29) | - |  | | | |
| **p-value** | - | - |  | - | - |  | - | - |  | - | - |  | - | - |  | | - | - |  |  | | | |
|  | | | | | | | | | | | | | | | | | | | |  | | | |
| **B** | **Meningeal infiltration** | | | **Vascular inflammation** | | | **Ventriculitis** | | | **Bleeding** | | | **Thrombosis** | | | | **Abscess** | | | **Total** | | | |
|  | **ST1**  **score^b^** | **ST6**  **score** | **p-value** | **ST1**  **score** | **ST6**  **score** | **p-value** | **ST1 score** | **ST6 score** | **p-value** | **ST1 score** | **ST6 score** | **p-value** | **ST1 score** | **ST6**  **score** | | **p-value** | **ST1 score** | **ST6 score** | **p-value** | **ST1**  **score** | **ST6 score** | **p-value** | |
| **Non-treatment model** | | | | | | | | | | | | | | | | | | | | | | | |
| **t=6** | 1 [1-2]^c^ | 1 [1-1] | - | 0 [0-0] | 0 [0-0] | - | 0 [0-0] | 0 [0-0] | - | 0,5 [0-1] | 0 [0-0] | 0,03 | 0 [0-0] | 0 [0-0] | | - | 0 [0-1] | 0 [0-0] | - | 2 [1-4] | 1 [0-1] | - | |
| **t=24** | 3 [3-3] | 3 [3-3] | - | 2 [2-2] | 2 [2-2] | - | 0 [0-3] | 1 [0-3] | - | 1 [0-1] | 1 [0-2] | - | 0 [0-1] | 0 [0-1] | | - | 0 [0-1] | 0 [0-1] | - | 7 [6-9] | 7 [5-9] | - | |
| **p-value** | <0,001 | <0,001 |  | <0,001 | <0,001 |  | - | 0,005 |  | - | - |  | - | 0,04 | |  | - | - |  | 0,001 | <0,001 |  | |
| **Treatment model** | | | | | | | | | | | | | | | | | | | | | | | |
| **t=16** | 3 [3-3] | 2 [2-3] | - | 2 [2-2] | 1 [1-1] | 0,01 | 1 [0-2] | 0 [0-1] | - | 1 [0-1] | 1 [1-1] | - | 0 [0-0] | 0 [0-0] | | - | 0 [0-0] | 0 [0-2] | - | 6 [5-8] | 6 [5-6] | - | |
| **t=24** | 3 [3-3] | 2 [2-3] | 0,004 | 2 [2-2] | 2 [1-2] | - | 2 [1-2] | 1 [0-2] | - | 2 [1-3] | 1 [1-1] | 0,01 | 0 [0-1] | 0 [0-1] | | - | 0 [0-0] | 0 [0-1] | - | 9 [8-12] | 7 [6-7] | 0,002 | |
| **p-value** | - | - |  | - | - |  | - | - |  | - | - |  | - | - | |  | - | - |  | - | - |  | |

**Additional file 3**. Histopathological scoring of brain tissue in listeria meningitis time point studies with ST1 and ST6. A. Results based on number of mice B. Results based on median pathology score.
