# Supplementary material for: Characterization of a Listeria monocytogenes meningitis mouse model
Source: J Neuroinflammation. 2018 Sep 7;15:257. doi: 10.1186/s12974-018-1293-3 (PMC6128981; doi:10.1186/s12974-018-1293-3)
Supplement: Supplementary file 5 — This table shows bacterial outgrowth in brain homogenate in mice infected with L. monocytogenes ST1 and treated with antibiotics during survival experiments (70 h post inoculation). Every bacterial count represents one mouse. (DOC 51 kb) [file 12974_2018_1293_MOESM5_ESM.doc]

**Additional file 5.** Bacterial outgrowth in brain homogenate in mice infected with ST1 and treated with antibiotics during survival experiments (70 hours post inoculation). Every bacterial count represents one mouse.

| **Inoculum size** | **Amoxicillin (mg/kg)** | **Additional gentamicin (mg/kg)** | **Bacterial outgrowth (CFU/mg)** |
| --- | --- | --- | --- |
| 10^9^ CFU/ml | 50 per 24 hours | - | 6 x 10^4^ |
|  | 100 per 24 hours | - | 2 x 10^5^ |
|  |  | - | 6 x 10^5^ |
|  |  | - | 2 x 10^4^ |
|  | 200 per 24 hours | - | 2 x 10^4^ |
|  |  | - | 1 x 10^6^ |
|  | 100 per 12 hours | - | 7 x 10^4^ |
| 10^8^ CFU/ml | 100 per 24 hours | - | 2 x 10^4^ |
|  |  | - | 2 x 10^5^ |
|  | 100 per 24 hours | 20 per 24 hours | 6 x 10^4^ |
|  |  |  | 7 x 10^4^ |

Abbreviations; CFU = colony forming units, mg = milligram, kg = kilogram, ml = microliter
