# Supplementary material for: Characterization of a Listeria monocytogenes meningitis mouse model
Source: J Neuroinflammation. 2018 Sep 7;15:257. doi: 10.1186/s12974-018-1293-3 (PMC6128981; doi:10.1186/s12974-018-1293-3)
Supplement: Supplementary file 7 — This table shows the brain and plasma levels of cytokines in mice infected with 109 CFU/ml L. monocytogenes ST1 or ST6 at time points 16 and 24 h and treated with 100 mg/kg/24 h amoxicillin after 16 h. (DOC 64 kb) [file 12974_2018_1293_MOESM7_ESM.doc]

| **Additional file 7.** Brain and plasma levels of cytokines in mice infected with 10^9^ CFU/ml *L. monocytogenes* ST1 or ST6 at time points 16 and 24 hours and treated with 100 mg/kg/24 hours amoxicillin after 16 hours. | | | | | | |
| --- | --- | --- | --- | --- | --- | --- |
|  | **Brain** | | | **Plasma** | | |
| **Cytokine^a^,**  **time^b^** | **ST1** | **ST6** | **P-value** | **ST1** | **ST6** | **P-value** |
| **IL-1β** |  |  |  |  |  |  |
| **16** | 2122 [1861 - 2939]^c^ | 2039 [1821 - 2660] | 0.76 | 262 [197 - 308] | 192 [128 - 206] | **0.04** |
| **24** | 1402 [1329 - 2043] | 1287 [1061 - 1992] | 0.79 | 199 [135 - 312] | 57 [126 - 180] | **0.04** |
| **IL-6** |  |  |  |  |  |  |
| **16** | 1928 [1776 - 2459] | 1396 [1025 - 1523] | **0.003** | 948 [765 - 1234] | 716 [556 - 1030] | 0.29 |
| **24** | 2664 [2248 - 3228] | 2221 [1821 - 3040] | 0.65 | 1000 [616 - 1280] | 1133 [1029 - 1547] | 0.29 |
| **IL-10** |  |  |  |  |  |  |
| **16** | NA^d^ | NA | - | 274 [232 - 285] | 235 [202 - 315] | 0.54 |
| **24** | NA | NA | - | 365 [248 - 427] | 197 [174 - 261] | **0.01** |
| **IL-17A** |  |  |  |  |  |  |
| **16** | 38 [28 - 43] | 21 [15 - 24] | **0.001** | 77 [71 - 94] | 54 [47 - 93] | 0.09 |
| **24** | 36 [24 - 40] | 22 [19 - 33] | 0.09 | 56 [48 - 63] | 53 [36 - 66] | 0.72  . |
| **TNF-α** |  |  |  |  |  |  |
| **16** | 1593 [1285 - 1939] | 512 [60 - 744] | **0.0004** | 501 [424 - 619] | 427 [298 - 522] | 0.25 |
| **24** | 1593 [1092 - 1937] | 817 [670 - 1543] | 0.30 | 427 [363 - 542] | 236 [210 - 329] | **0.04** |
| **IFN-γ** |  |  |  |  |  |  |
| **16** | 29 [26 - 45] | 19 [17 - 23] | **0.01** | 66 [55 - 92] | 62 [58 - 74] | 0.36 |
| **24** | 33 [22 - 39] | 19 [18 - 24] | 0.12 | 108 [81 - 127] | 61 [46 - 88] | 0.29 |

^a^ Cytokines expressed in pg/ml, ^b^ time in hours, ^c^ median level and interquartile range, ^d^ NA not applicable; all values under the lower limit of detection
